# MetALD: decoding the evolution of steatotic liver disease nomenclature and implications for clinical practice and beyond

**DOI:** 10.1093/gastro/goag006

**Published:** 2026-02-20

**Authors:** Farinaz Ghodrati, Ashley B Zhang, Nadim Mahmud

**Affiliations:** Department of Medicine, Perelman School of Medicine, University of Pennsylvania, Philadelphia, PA, United States; Department of Medicine, Perelman School of Medicine, University of Pennsylvania, Philadelphia, PA, United States; Division of Gastroenterology and Hepatology, Perelman School of Medicine, University of Pennsylvania, Philadelphia, PA, United States; Department of Medicine, Corporal Michael J. Crescenz VA Medical Center, Philadelphia, PA, United States; Leonard Davis Institute of Health Economics, University of Pennsylvania, Philadelphia, PA, United States; Center for Clinical Epidemiology and Biostatistics, Department of Biostatistics, Epidemiology & Informatics, Perelman School of Medicine, University of Pennsylvania, Philadelphia, PA, United States

**Keywords:** MetALD, MASH, MASLD, SLD, ALD, liver transplant

## Abstract

The steatotic liver disease (SLD) landscape has seen a paradigm shift in recent years with a revitalization of the nomenclature following a multi-society Delphi consensus. The terms metabolic dysfunction-associated steatotic liver disease (MASLD) and metabolic dysfunction-associated steatohepatitis (MASH) were introduced to address several of the challenges and limitations associated with the former terminology. By transitioning away from stigmatizing and ambiguous terms, the nomenclature has adopted inclusionary language that emphasizes the underlying risk factors that drive disease progression and are accompanied by distinct diagnostic criteria. With SLD prevalence steadily increasing over the past few decades, affecting over 30% of the global population, accurate classification of the spectrum of conditions that fall under this overarching term is essential. Most importantly, the introduction of combined metabolic and alcohol-associated liver disease (MetALD) as a novel subclassification of SLD has shifted the diagnostic approach, raised awareness of disease prevalence, and paved the way for therapeutic management and multidisciplinary approaches to patient care. By recognizing the distinct clinical entity that is MetALD and the synergistic interplay between the cardiometabolic risk factors and alcohol use, clinicians are better equipped to effectively care for this patient population. In this review, we aim to discuss the catalysts for the SLD nomenclature changes, the dynamic nature of its subclasses, the natural history and disease burden, and the implications for clinical practice and research, with a particular focus on MetALD.

## Introduction

Chronic liver disease (CLD) refers to progressive liver injury for greater than 6 months and includes a broad range of etiologies ranging from metabolic disorders to alcohol use [1]. With time, CLD leads to accumulated hepatic fibrosis and ultimately cirrhosis [1]. Globally, CLD affects more than 30% of the population and remains one of the most frequent causes of death, both worldwide and in the United States (US) [2]. In 2018, the Center for Disease Control and Prevention reported 4.5 million adults were diagnosed with liver disease, equating to 1.8% of the US population [3]. Most recently, in 2023, CLD and cirrhosis accounted for 15.6 deaths per 100,000 population in the US, alone, making it the ninth-leading cause of mortality in the nation [3].

The most common historic causes of CLD and cirrhosis include chronic viral hepatitis, alcohol-associated liver disease (ALD), non-alcoholic fatty liver disease (NAFLD), and non-alcoholic steatohepatitis (NASH). Other causes can include underlying genetic etiologies (e.g. hereditary hemochromatosis, Wilson disease), autoimmune disease (e.g. autoimmune hepatitis, primary biliary cholangitis, primary sclerosing cholangitis), and drugs and toxins, among other less common etiologies [1].

Within the spectrum of CLD, conditions characterized by hepatic steatosis, such as NAFLD and NASH, are among the most prevalent and fastest-growing worldwide. In 2019, NAFLD had a global prevalence of 37%–38% and a North American prevalence of 35%–37%, while NASH has a prevalence of approximately 5% [2, 4]. Moreover, the global prevalence of NAFLD has been rising at an annual rate of 0.7%, leading to an increase from 21.9% in 1991 to 37% in 2019 [4]. Across the period of 1990 to 2019, NAFLD had a pooled mortality rate of 12.60 per 1,000 person-years [4]. Given their significant public health impact, experts have recognized the need for terminology that accurately reflects underlying biology, captures overlapping etiologies, and avoids stigma. While NAFLD has been widely used in the field for the past 45 years, it has long been thought that the term “non-alcoholic” does not accurately depict the etiology of the disease and trivializes the importance of cardiometabolic risk factors (CMRFs); the term “fatty” has been considered to be stigmatizing [5, 6]. Additionally, growing recognition that many patients classified as having NAFLD or ALD actually have features of both has further fueled calls for revision. As such, a proposal was set forth in 2020 to revise steatotic liver disease (SLD) nomenclature and in 2023, the American Association for the Study of Liver Disease (AASLD), the European Association for the Study of the Liver (EASL), and the Liver-Asociación Latinoamericana para el Estudio del Hígado (ALEH) released a multi-society Delphi consensus statement proposing a change in nomenclature for the conditions formally known as NAFLD and NASH, as well as creating a novel classification for overlapping metabolic and alcohol-related SLD [6, 7]. With endorsements from more than 70 major societies worldwide, this catalyzed the transition to the term SLD as the umbrella term encompassing various etiologies of steatosis [6].

The overarching SLD category encompasses metabolic dysfunction-associated steatotic liver disease (MASLD) and its inflammatory subtype, metabolic dysfunction-associated steatohepatitis (MASH), which replace NAFLD and NASH, respectively, as well as ALD and other less common steatosis etiologies. In addition, the 2023 Delphi consensus introduced combined metabolic and alcohol-associated liver disease (MetALD) as a newly defined subcategory that exists on a spectrum between MASLD and ALD, accounting for both MASLD-defining CMRFs, and alcohol intake [6]. Experts supported establishing MetALD as distinct from MASLD given that alcohol use has an independent pathogenic role and important prognostic implications in patients with CMRFs [6]. In addition to more comprehensively capturing a nuanced spectrum of common liver diseases, the novel SLD classification creates opportunities for targeted research to better define the natural history, identify disease biomarkers, and develop tailored therapeutic approaches for this patient population [6].

This review highlighted the evolution of SLD nomenclature with a focus on MetALD and discussed the impact these changes have had in clinical practice, research, policy, and beyond.

## Methodology

We conducted a targeted literature review using PubMed, Embase, and Web of Science to identify publications on SLD, MASLD, MetALD, and ALD. Searches extended from first available dates in the respective databases through 8/2025 and used combinations of keywords and medical subject headings (MeSH) terms including but not limited to: steatosis, liver disease, history of liver disease, metabolic dysfunction, alcohol intake, prevalence, mortality, biomarkers, and therapy. Studies were reviewed for relevance to this review, and additional references were identified from bibliographies of key reviews and society practice guidelines (e.g. AASLD, EASL). Priority was given to multi-center prospective cohorts, large administrative datasets, randomized clinical trials, and high-quality consensus statements. Evidence was synthesized narratively, emphasizing consistency across studies and highlighting remaining gaps.

## Evolution of nomenclature and definitions

### From the nineteenth century to NAFLD/NASH

Clinical and pathological records of fatty liver features date back as early as the nineteenth century. In 1836, Thomas Addison described cases of liver degeneration related to excessive alcohol intake [8]. In 1843, Cecil Watson described the gross anatomy of fatty liver as “inordinately large, of a smooth, uniform color and appearance” [9]. Following this, Goerge Budd and Rudolf Virchow both described the microscopic appearance of fatty liver in 1857 and 1858, respectively [10]. Entering the twentieth century, the pathogenesis of fatty liver slowly emerged and this disease was recognized as a heterogenous condition that exists on a spectrum of severity [10].

In 1980, Jurgen Ludwig and colleagues coined the term NASH after describing findings over a 10-year period in 20 patients, predominately women, with obesity and/or adult-onset diabetes who had histologic evidence suggestive of alcohol hepatitis on biopsy but no history of extensive alcohol use [11]. Morphologically, the pattern of liver injury involved lobular inflammation and fatty changes [11]. It was not until 1986 that Fenton Schaffner introduced the term NAFLD, which became the encompassing term to represent the spectrum of observed pathology, which ranged from hepatic steatosis, to the lobular inflammation and Mallory hyaline seen in NASH [10, 12].

### Transition from NAFLD/NASH to MASLD/MASH

In the modern era, NAFLD has been used as an umbrella term to refer to patients with steatosis in 5% or greater of hepatocytes without a readily identifiable cause of steatosis, such as monogenic disorders, starvation, medications, or significant alcohol intake–defined as less than 30 g/day for males and less than 20 g/day for females [13, 14]. NASH was defined as hepatic steatosis accompanied by inflammation and cellular injury with or without the presence of fibrosis [14]. The impetus for the nomenclature change was to rectify several of the limitations associated with the terms NASH and NAFLD, including the lack of emphasis on the fundamental cause of the disease, the exclusionary basis of the diagnosis, the need for clear diagnostic criteria, as well as the use of potentially stigmatizing language [6, 15, 16].

Following a global Delphi consensus process involving national hepatology and endocrinology societies, as well as patient advocacy groups, a novel nomenclature system was introduced [6, 16]. SLD was chosen as the overarching term, encompassing MASLD, MetALD, ALD, and specific SLD etiologies, such as monogenic diseases (e.g. Wilson disease and inborn errors of metabolism), drug-induced liver injury (DILI), miscellaneous [e.g. viral causes including hepatitis C virus (HCV) and human immunodeficiency virus (HIV)], and lastly cryptogenic SLD [6, 16].

MASLD is defined as hepatic steatosis in addition to one or more CMRFs in the absence of other known cause of steatosis [6, 16]. CMRFs were chosen to align with factors linked to insulin resistance and those well-established within the framework of cardiovascular disease, which broadly include obesity, diabetes, hypertension, and dyslipidemia [6, 16]. In particular, five CMRFs were selected: i) Body mass index (BMI) ≥ 25 kg/m^2^ [23 in Asians] or weight circumference > 94 cm (males) or 80 cm (females) or ethnicity adjusted equivalents; ii) Fasting serum glucose ≥ 100 mg/dL or 2-hour post-load glucose levels ≥ 140 g/dL or Hgb A1c ≥ 5.7% or type 2 diabetes (T2DM) or treatment for type 2 diabetes; iii) Blood pressure ≥ 130/85 mmHg or specific antihypertensive drug treatment; iv) Plasma triglycerides ≥ 150 mg/dL or lipid lowering treatment; v) Plasma high-density lipoprotein (HDL)-cholesterol ≤ 40 mg/dL (males) and ≤ 50 mg/dL (females) or lipid-lowering treatment [6, 16].

Although NAFLD and MASLD have distinct definitions, emerging data since the name change have demonstrated that there is close diagnostic concordance between the two [16, 17]. This was by design, in order to preserve the wealth of existing literature on the natural history and biomarkers of the disease [6]. There are a number of studies highlighting this concordance [17]; a study of the European NAFLD registry found that 98% met the MASLD criteria [18], a Hong Kong study found a similar prevalence of the two in a random population sample of 1,016 individuals with proton-magnetic resonance spectroscopy [19], a French study found more than 98% of their 2,187 NAFLD patients fulfilled MASLD criteria [20], a Swedish study on 1,333 patients found that more than 99.5% of NAFLD patients met MASLD criteria and argued that the two therefore have identical natural history [21], and lastly a US study of 6,429 NAFLD patients showed 99.8% fulfilled MASLD criteria [22].

NASH was replaced with MASH, which refers to MASLD meeting histologic criteria of steatohepatitis. Conversely, MASLD criteria not meeting steatohepatitis is denoted as metabolic dysfunction–associated steatotic liver (MASL), mirroring how non-alcoholic fatty liver (NAFL) described NAFLD that did not meet NASH criteria [6, 16].

With these changes in terminology, it is thought that affirmative as opposed to exclusionary language and criteria will help optimize patient communication in order to better convey the underlying cause of the disease and in turn, the therapeutic and lifestyle changes suggested [6]. Similarly, these changes are beneficial to healthcare providers and enhance identification of patients with known metabolic and cardiovascular risk factors who may be at risk [6].

### MetALD and ALD diagnostic criteria

MetALD is defined as the triad of hepatic steatosis, CMRFs, and an average daily alcohol intake of 20–50 g in females and 30–60 g in males or weekly alcohol intake of 140–350 g in females and 210–420 g in males [6, 16, 23]. Correspondingly, ALD denotes alcohol intake >350 g/week for females and >420 g/week for males, irrespective of CMRFs [6, 16]. These thresholds for alcohol consumption were established by the Delphi consensus as part of the recognition of MetALD as a new category [6, 23].

**Figure 1:**
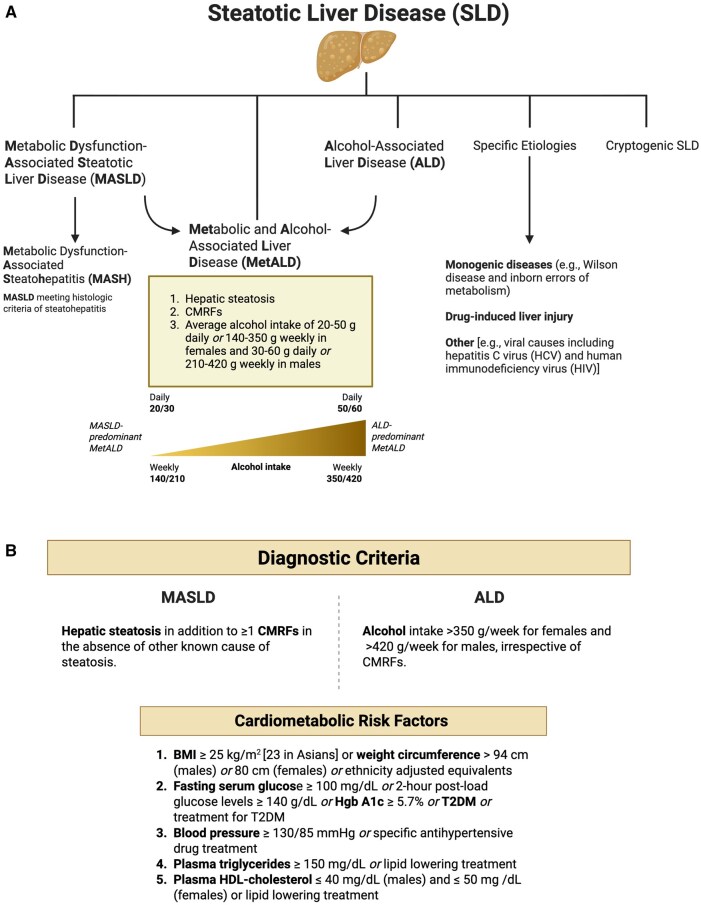
SLD classification and diagnostic criteria. (A) Spectrum of SLD phenotypes and diagnostic criteria for MetALD. (B) Diagnostic criteria for MASLD and ALD, and the criteria for CMRFs. ALD, alcohol-associated liver disease; BMI, body mass index; CMRFs, cardiometabolic risk factors; HCV, hepatitis C virus; HDL, high density lipoprotein; HIV, human immunodeficiency virus; MASH, metabolic dysfunction-associated steatohepatitis; MASLD, metabolic dysfunction-associated steatotic liver disease; MetALD, metabolic and alcohol-associated liver disease; SLD, steatotic liver disease; T2DM, type 2 diabetes mellitus.

Despite clear cutoffs set forth regarding MetALD diagnostic guidelines, there exists a spectrum of disease, spanning from MASLD-predominant to ALD-predominant disease [6, 16]. Additionally, alcohol use as well as CMRFs are dynamic variables changing over time [24], which leaves room for diagnostic ambiguity in SLD subclasses. A schematic overview of the novel SLD phenotypes and diagnostic criteria is shown in Figure 1.

Key Points

•  In 2023, following a Delphi consensus process, the terms NAFLD and NASH were replaced by new terminology: MASLD and MASH, respectively.
•  MetALD emerged as a new subclassification of SLD, defined as the triad of hepatic steatosis, CMRFs, and an average weekly alcohol intake of 140–350 g in females and 210–420 g in males.

## Key controversies surrounding the novel nomenclature

### Dynamic nature of SLD subclasses

Given the dynamic nature of alcohol consumption and its impact on cardiometabolic risk, a prospective observational study of more than 1,000 Danish individuals aimed to assess the longitudinal robustness of the subclasses under the overarching SLD schema [24]. Not only can alcohol intake evolve significantly over an individual’s lifetime, the impact of alcohol on related factors including exercise, diet, and sleep, affects CMRFs as well as the presence of steatosis and consequently, the subclassification of SLD [24]. Importantly, CMRFs themselves are dynamic over time and may improve or worsen with changes in weight, lifestyle, medical therapy, and alcohol use. As a result, individuals may transition between SLD subclasses across the disease course, highlighting that these categories represent evolving phenotypic states rather than fixed diagnoses.

### Limitations and unintended consequences of the SLD classification

Although studies have shown that there exists a substantial under-reporting of alcohol intake in 10%–55% of patients diagnosed with MASLD, there is a scarcity in data examining the potential over-reporting and consequent overdiagnosis of MetALD and/or ALD [25]. Some patients may be misclassified as having MetALD when alcohol exposure is intermittent, inaccurately quantified, or clinically insignificant. For example, a patient with significant alcohol use at a discrete time point who subsequently stops drinking—but in whom this history is carried forward in the medical record—may be inappropriately labeled as having MetALD or ALD. Such overdiagnosis may lead to inappropriate labeling, stigmatization, misapplied management pathways, or unnecessary exclusion from MASLD-directed therapies and clinical trials.

In the aforementioned Danish study, when the SLD diagnosis of participants was reassessed 2 years after the initial assessment, more than 30% of participants had switched classes from baseline with the prevailing reasons being changes in alcohol use and no longer fulfilling SLD criteria [24]. MASLD remained the most stable with a 38% change, while MetALD and ALD had the largest relative change of more than 60% [24]. This supports the concept that alcohol consumption leading up to an SLD diagnosis may not serve as an accurate representation of future alcohol consumption following diagnosis [24]. This has substantial consequences, especially given that it is broadly accepted that the pattern of alcohol use following a diagnosis holds prognostic implications [24].

The definition of ALD implies that sufficient alcohol use mandates a classification of ALD, regardless of the number of CMRFs present. In one study, 92% of individuals meeting ALD criteria had a minimum of one CMRF present, again highlighting the overlap of contributing factors to this disease paradigm [26]. A recent national cohort study of patients with SLD cirrhosis receiving care in the Veterans Health Administration (VHA) system, where annual alcohol screening tools are universally applied, found that within those meeting ALD criteria, 25% had four CMRFs and 38% had five CMRFs [27]. The authors used multiple algorithms with different thresholds of alcohol use and CMRFs to classify MASLD, MetALD, and ALD, and demonstrated that SLD classification is highly sensitive to specific thresholds with respect to alcohol intake and CMRFs [27]. Classifying patients with any CMRF and alcohol use as MetALD led to a distribution of 34.5% MASLD, 64.2% MetALD and 1.1% ALD while defining ALD based solely on excessive alcohol use yielded 4.7% as MetALD and 60.6% as ALD, and lastly when only those with heavy alcohol use and high CMRF burden where classified as MetALD a more balanced distribution of 35.6% MetALD and 29.6% ALD was found [27]. With the latter classification schema, it is also noteworthy that outcomes of major adverse cardiovascular events (MACE) and alcohol use disorder (AUD)-related hospitalizations were intuitively aligned with SLD categories; that is, a mix of these outcomes was observed in MetALD, whereas MASLD demonstrated predominant MACE outcomes and ALD predominant AUD-related hospitalization outcomes [27]. These studies raise the question as to whether ALD is the appropriate classification for patients with concomitant heavy alcohol use and substantial CMRF burden, or if MetALD would be a more accurate diagnosis for a subset of individuals in this cohort.

### MetALD: a distinct entity

MetALD is recognized as a distinct entity given that metabolic disease heightens the risk of steatohepatitis and fibrosis in ALD and, in a similar fashion, alcohol use raises the risk of fibrosis in MASLD or MASH [28]. The MetALD nomenclature highlights the recognition that both CMRFs and alcohol intake not only contribute to liver disease progression, but that there may also be a synergistic effect [16, 29]. This clear acknowledgement of the parallel existence of alcohol use and CMRFs also emphasizes the significance of addressing both factors in order to better care for patients with liver disease [16].

Studies have highlighted the interplay of alcohol intake and metabolic risk factors in liver disease development and progression, with both alcohol damage and metabolic injury sensitizing the liver to the harmful effects of the other [29–31]. Clinical and mechanistic studies appear to suggest an additive impact on liver disease when it comes to the interaction of metabolic risk and alcohol intake, meaning that in the presence of both factors, the disease risk is amplified beyond that of the combined independent risks [31].

### Operational challenges

There are also logistical obstacles that arise with a new subclassification of disease. To date, there is no ICD-10 code for MetALD which presents challenges for epidemiologic monitoring through electronic medical records databases [32]. Another medical documentation challenge with MetALD lies within accurate reporting of alcohol intake due to both changing consumption patterns over a patient’s lifetime as well as under- and over-reporting, as previously mentioned [25]. Gathering a reliable history of alcohol consumption is riddled with challenges given the various social, cultural, psychological, and religious factors at hand that can affect disclosure of drinking habits [25]. Stigma associated with alcohol use can deter patients from being forthcoming as they perceive having alcohol as the main contributor of disease to be socially unacceptable and fear judgment from clinicians and being treated differently in healthcare settings [25]. On the other hand, those with metabolic risk factors as the driver of their liver disease might perceive their alcohol consumption as negligible and not worth disclosing despite even small quantities of alcohol contributing to disease progression [25]. Lastly, recall bias serves as another obstacle when obtaining an alcohol use history [25].

Furthermore, standardized approaches for assessing and documenting alcohol use remain limited across most health systems. One notable exception is the VHA, which requires providers to administer the Alcohol Use Disorders Identification Test–Consumption (AUDIT-C) annually [33]. This structured approach highlights the gap in routine alcohol use assessment elsewhere, where inconsistent practices compound the challenges of accurately capturing alcohol-related contributions to liver disease.

Key Points

•  Because an individual’s CMRFs and use of alcohol can evolve over time, the SLD subclassifications of MASLD, MetALD, and ALD exist on a dynamic spectrum.
•  Adopting the new nomenclature of MetALD acknowledges the synergistic effects of alcohol consumption and CMRFs on the progression of liver disease.
•  Under- or over-reporting of alcohol consumption may lead to misclassification of SLD diagnosis; future work is needed to improve accurate assessment and reporting.
•  Within the current administrative coding system, there is no ICD-10 code for MetALD which presents a limitation for disease monitoring and observational research.

## Epidemiology and natural history

### Prevalence of SLD subclasses

SLD prevalence has been on the rise over the last few decades with analyses of large databases, such as the US National Health and Nutrition Examination Survey (NHANES) and the UK Biobank, estimating a global prevalence of 30%–40% [25, 34, 35]. Alcohol is estimated to contribute to a marginal proportion of global SLD cases with ALD and MetALD comprising 1% and 2%–3% of cases, respectively [25, 34]. As such, MASLD accounts for the remaining 90% of patients. Based on recent NHANES data, the age-standardized prevalence of SLD in the US population is 37.08%, of which 32.42% is attributed to MASLD, 2.20% to MetALD and 1.29% to ALD [35]. MetALD affects an estimated 17 million adults in the US [32].

While MASLD is commonly associated with metabolic syndromes and obesity, the prevalence of lean MASLD, defined using a BMI threshold of <25 kg/m^2^ (<23 kg/m^2^ in non-Hispanic Asians), is approximately 9.3% using age-adjusted data from NHANES [36, 37]. The prevalence of lean MetALD and ALD has been reported to be 1.3% and 1.0%, respectively [36, 37]. In particular, non-Hispanic Asians and Hispanics demonstrated a higher prevalence of lean MASLD whereas non-Hispanic Caucasians were at a higher risk for lean MetALD and ALD [36]. Given the recent introduction of the MetALD classification, epidemiologic data on lean MetALD remain limited, particularly with respect to longitudinal outcomes and disease progression [36].

It is important to note that 90% of North American and European populations consume alcohol, with 1 in 4 individuals reporting regular binge drinking habits, and 1 in 10 individuals partaking in harmful use of alcohol [25, 38]. According to the World Health Organization (WHO) binge drinking is defined as drinking ≥60 g of alcohol on one occasion and harmful use as drinking that causes adverse health and social consequences [38]. The discrepancy between the reported prevalence of MetALD and ALD versus alcohol use in the general population suggests substantial underreporting of alcohol use in the above studies and therefore calls into question the low rates of alcohol-related SLD. In an epidemiologic study of patients listed for liver transplantation that incorporated manual adjudication of medical charts, the distribution of patients with SLD was 38.2% MASLD, 22.5% MetALD, and 39.4% ALD [39]. While transplant evaluation cohorts differ from population-based samples because they are enriched for advanced liver disease and referral-based selection, and therefore cannot be directly compared to population prevalence estimates, the marked enrichment of MetALD and ALD in this setting likely reflects more intensive and longitudinal assessment of alcohol use, providing indirect evidence of underreporting in population-based studies.

Consistent with this interpretation, multiple independent lines of evidence demonstrate substantial under-reporting of alcohol intake among patients with a presumed MASLD diagnosis [25]. An Austrian study found that 29% of MASLD patients may more accurately be labeled as MetALD based on alcohol biomarkers [40], a Swedish study investigating ICD-10 codes showed that 17% of those with the MASLD diagnosis had prior or concurrent diagnoses of AUD or ALD [41], and a US observational study comparing self-reported alcohol consumption to phosphatidylethanol (PEth) levels found 58% of participants under-reported alcohol use [42]. Similar patterns have also been shown in the rigorous clinical trial settings; for instance, in the MAESTRO-NASH trial, despite strict exclusion criteria for self-reported alcohol use, roughly 10% of participants were positive for biomarkers suggestive of MetALD [25, 43, 44].

Further evidence of this underdiagnosis can be observed by analyzing the mismatch between liver-related outcomes and prevalence of SLD subclasses [45]. Although MASLD has a 10-fold higher prevalence, MetALD and ALD share a much higher burden of hospitalizations, decompensations, and liver-related mortality experienced by patients [24, 46, 47]. More than 50% of all cirrhosis-related hospitalizations in Germany from 2005 to 2018 were due to alcohol-related cirrhosis while only 3% was accounted for by MASLD [45, 46]. In a UK study of more than 1,200 patients with a hospitalization due to decompensated cirrhosis, alcohol-related liver disease comprised 75% of admissions while only 14% were related to MASLD [47].

Underestimation of ALD and MetALD has broad implications for the understanding, accurate classification, research, and management of these diseases [25]. The discrepancies highlighted above underscore the need for standardized, high-fidelity alcohol phenotyping—pairing validated questionnaires (e.g. AUDIT-C) with objective biomarkers [e.g. PEth/ethylglucuronide (EtG)], and capturing binge patterns and lifetime exposure—to reduce misclassification when estimating SLD subclass prevalence and assigning phenotypes [48].

### Natural history and burden of disease

ALD and MASLD have distinct clinical courses with ALD being more aggressive and harboring a worse prognosis, despite some overlap in pathological attributes of steatosis and steatohepatitis [32, 49]. As compared to MASLD, ALD usually presents at more advanced stages of disease and is associated with higher risk of developing cirrhosis, complications of cirrhosis, and hepatocellular carcinoma (HCC) [32, 49]. Liver-related deaths for MetALD and/or ALD was found to be 45% following diagnosis while only 4% for biopsy-proven MASLD [45]. MetALD is associated with a higher risk of both cirrhosis and HCC when compared to MASLD [50].

Not only does liver disease portend a higher risk of HCC, but also, individuals with liver disease are at risk of developing extrahepatic cancers, such as pancreatic, gastric, esophageal, and most notably colorectal cancer given that obesity and alcohol consumption are risk factors in both conditions [51]. In a nation-wide population study conducted in Japan, the incidence of colorectal cancer was examined in individuals with MASLD, MetALD, or ALD, compared to individuals with no known liver disease. The findings revealed that both the 5- and 10-year cumulative incidence of colorectal cancer was highest in ALD, followed by MetALD and MASLD [52].

An NHANES study of 9,939 participants followed for up to 27 years found that survival followed an expected pattern and was highest in those without SLD followed by individuals with MASLD, MetALD, and ALD [26]. Compared to those without SLD, having MASLD, MetALD, or ALD was linked to a 16%, 33%, or 75% higher mortality, respectively [26]. However, using MASLD as a reference, the mortality of MetALD patients was not significantly higher than MASLD patients while ALD patients had a 57% higher mortality compared to MASLD [26].

A prospective cohort study found that the risk of hepatic decompensation and all-cause mortality increases progressively from MASLD to MetALD to ALD, with the latter demonstrating the highest risk, showing a statistically significant difference between MASLD and MetALD mortality, unlike the NHANES study above [53]. Similar to this, a retrospective cohort study of US veterans found that those with MetALD and ALD had a higher risk of adverse liver outcomes and all-cause mortality and similar risks of MACE as compared to MASLD patients [54].

To further evaluate and compare all-cause mortality in these populations, Li *et al.* conducted the first reported meta-analysis examining 164,694 individuals to determine the association between MetALD and all-cause mortality compared to individuals with MASLD and also those without SLD [55]. This meta-analysis revealed that MetALD is an independent risk factor for all-cause mortality [55]. Furthermore, when compared to MASLD, MetALD presents an additional risk factor for all-cause mortality as a result of increased alcohol consumption and the synergistic effect it has combined with CMRFs in this patient population. [55].

### Trends in liver transplantation

Since 1980, the worldwide leading cause of liver transplantation was HCV [56]. With the introduction of direct-acting antiviral agents in the mid-2010s, there has been a global shift observed in the underlying etiology of liver disease driving transplantation [56, 57]. In the US, the Organ Procurement and Transplantation Network (OPTN) and Scientific Registry of Transplant Recipients (SRTR) reported a decrease in HCV-related liver transplantations from 31.2% to 8.6% from 2009 to 2022 [58]. With the increase in obesity and cardiometabolic disease, MASLD now represents the leading indication for liver transplant in HCC patients and the second most common underlying etiology in non-HCC patients [56]. Furthermore, with the new subclassification of MetALD, which reportedly affects over 17 million individuals in the US, albeit with the true prevalence likely much higher as discussed earlier, there is a growing need to incorporate this new SLD entity into liver transplantation data structures and statistics [39].

Using the United Network for Organ Sharing (UNOS) national registry, a recent study developed and validated an algorithm to identify novel SLD phenotypes and to assess trends in liver transplant rate and outcomes [39]. Within MetALD patients, the authors found a 2.9-fold (from 5.6% to 16.2%) increase in waitlist registration rate and a 3.3-fold (from 4.8% to 15.9%) increase in liver transplant rate over a 20-year period between 2002 and 2022 [39]. This trend is on par with the concomitant increase in alcohol use and obesity both nationally and worldwide in recent years [39]. Among patients waitlisted for liver transplantation, the SLD phenotypes are the most common among all etiologies of liver disease by far, with ALD most common, followed by MASLD and then MetALD [39].

Key Points

•  In the US, the prevalence of SLD is approximately 37%, with MASLD accounting for ∼32%, MetALD ∼2%, and ALD ∼1%. MetALD is estimated to affect 17 million adults in the US.
•  The prevalence for MetALD is likely underestimated due to underreporting of alcohol intake in patients with presumed MASLD.
•  MetALD is an independent risk factor for all-cause mortality.
•  MASLD is currently the leading indication for liver transplant in patients with HCC.

## Implications for clinical practice

### Diagnostic and prognostic challenges: overlap and attribution

As noted, diagnosis of SLD subclasses requires accurate alcohol intake documentation by both the patient and medical professionals; however, self-reports on alcohol use are sensitive to recall bias and not always accurate [32]. The current clinical landscape lacks tools to quantify alcohol consumption more objectively [32]. Even PEth levels are limited by their window of detection being restricted to alcohol use in the past 6–8 weeks, posing challenges in the diagnosis of MetALD [32].

Current US and European guidelines recommend using a stepwise approach with the fibrosis-4 index (FIB-4) followed by vibration-controlled transient elastography (VCTE) to stage fibrosis and risk stratify MASLD patients [14, 23, 59–61]. However, another diagnostic challenge arises pertaining to the use of non-invasive fibrosis assessment scores in MetALD patients given that, unlike MASLD and ALD [62, 63], clear cutoffs have not been defined [32]. Non-invasive fibrosis screening tools, such as FIB-4, are known to have suboptimal diagnostic performance in ALD, which is hypothesized to be due to the different mechanisms of disease, variety of the conditions under the ALD umbrella, such as alcohol-related hepatitis, as well as the independent effects of alcohol on the nature of transaminase elevation and platelet count reduction, which confound scores such as the FIB-4 [64]. The utility of such tools in MetALD patients is therefore unclear given the plausible impact of moderate alcohol consumption in this group [64].

Despite these theoretical concerns, several recent studies suggest that risk stratification algorithms used in MASLD may in fact translate adequately to patients with MetALD [64, 65]. A cross-sectional study of more than 600 US participants found that the stepwise risk stratification approach used in MASLD with FIB-4 and VCTE performed well in detecting advanced fibrosis in MetALD patients with a false negative rate of 2%, compared to magnetic resonance (MR) elastography as the gold standard [65]. Advanced fibrosis was defined as liver stiffness measurement (LSM) ≥ 3.14 kPa on MR elastography and LSM ≥ 7.6 kPa on VCTE [65]. Similarly, a Korean cross-sectional SLD study of 7,918 participants with a MetALD prevalence of 5.8% found that FIB-4 had a comparably high diagnostic accuracy in identifying advanced fibrosis in both MASLD and MetALD patients, defined as LSM above 3.6 kPa on MR elastography [64]; diagnostic accuracy in ALD was inferior to other SLD subtypes.

Other tools, such as the steatosis-associated fibrosis estimator (SAFE), have also been studied in this context. A US study found that the SAFE score outperformed the FIB-4 score in stratifying patients with MASLD, MetALD, and ALD by their risk of long-term mortality, consistent with the SAFE score’s design for detecting earlier-stage fibrosis as compared with FIB-4’s focus on advanced fibrosis [26].

### Lifestyle and behavioral interventions

Lifestyle interventions, including diet and exercise, are first-line treatment recommendations for MASLD patients, with AASLD guidance recommending a goal weight loss of at minimum 5% and ideally ≥10% [28, 32]. While these strategies are likely applicable to MetALD, it is important to note that the vast majority of supporting evidence comes from MASLD/MASH studies, and dedicated research in MetALD is lacking [28, 32]. Weight loss management is multifaceted, encompassing dietary and nutritional changes, exercise, pharmacological interventions, and bariatric surgery in selected individuals [32]. Dietary modifications should emphasize a caloric deficit, with macronutrient intake limited to 50%–60% from carbohydrates and 20%–25% from lipids [28]. Additionally, routine aerobic exercise in 30–60 minute intervals 3–4 times weekly for 4–12 weeks has been linked to improvement in liver fat content even in absence of weight loss [28]. Bariatric surgery should be considered in eligible obese patients with MASLD/MASH. The eligibility criteria in short comprises patients with BMI ≥ 40 kg/m^2^, patients with BMI ≥ 35 kg/m^2^ with one or more severe obesity-related complication including but not limited to T2DM, hypertension, or MASLD, and lastly patients with BMI 30–34.9 kg/m^2^ and uncontrolled T2DM despite optimal lifestyle and medical therapy (of note BMI should be adjusted for ethnicity) [66]. In MetALD, however, the role of bariatric surgery remains uncertain due to the need for preoperative alcohol cessation, the potential for alcohol-induced liver injury following surgery, and evidence linking Roux-en-Y gastric bypass to new-onset or worsening AUD [67, 68]; further research is therefore needed before clear recommendations can be made [28, 32].

### Role of pharmacologic treatments

There have been over 1,200 clinical trials addressing SLD, with over 500 active or completed trials specifically focused on patients with MASLD or MASH [28]. Given the recent classification of MetALD, trial data in this specific population are currently limited. Pharmaceutical development for MetALD is further complicated by the biological and logistical effects of ongoing alcohol consumption. These include risks of nutritional deficiencies, hypoglycemia, hepatotoxicity, and altered drug metabolism via cytochrome P450 enzymes, as well as adherence and follow-up challenges, often compounded by comorbid psychiatric illness [28]. Enrolling participants with AUD in clinical trials is also challenging because most studies require sustained abstinence, which reduces eligibility and may bias enrollment toward individuals with less severe AUD, thereby limiting generalizability. Accordingly, therapeutic considerations in MetALD currently rely largely on extrapolation from MASLD and ALD trials, informed by biological plausibility and limited subgroup data, rather than direct evidence from MetALD-specific randomized studies.

Resmetirom, a selective thyroid hormone receptor beta agonist, is the first Food and Drug Administration (FDA)-approved therapy for adults with MASH with moderate to advanced fibrosis (F2-F3). Its approval was based on phase 3 trial data demonstrating histologic improvement, including resolution of MASH and reduction in fibrosis stage [69]. Although no medications are currently approved specifically for MetALD, agents developed for MASLD, such as resmetirom, cannot currently be recommended for MetALD based on direct evidence, but may plausibly have similar effects given the frequent coexistence of CMRFs in both conditions. For example, in the MAESTRO-NASH trial, which assessed the efficacy of resmetirom in patients with MASH, approximately 10% had possible MetALD and achieved response rates for steatohepatitis resolution (29%–35%) and fibrosis improvement (30%–35%) similar to those observed with MASLD/MASH without significant alcohol intake [43, 44].

Several therapeutics are in phase 3 trials for SLD subclasses. Among these, the most promising drug classes for potential use in MetALD are glucagon-like peptide-1 (GLP-1) receptor agonists, dual GLP-1/gastric inhibitory polypeptide (GIP) receptor agonists, and fibroblast growth factor-21 (FGF-21) analogs which may address both alcohol consumption and metabolic dysfunction [28, 32, 48]. GLP-1 and dual GLP-1/GIP receptor agonists, currently approved for T2DM and obesity, have been associated in most preclinical and clinical studies with reduced alcohol intake, raising the possibility of a role of AUD agonists can yield a reduction in alcohol intake, raising the possibility of their utilization in AUD management [32, 70, 71]. In addition, GLP-1/GIP analogs have shown histologic improvements in MASLD with significant reduction in weight, including reductions in steatohepatitis and fibrosis [32, 71–74]. This dual activity to address CMRFs and potential to reduce alcohol consumption suggests a strong rationale for future study of GLP-1 and GLP-1/GIP analogs in patients with MetALD [32, 70, 71]. Similarly, FGF-21, a key metabolic regulator expressed in the liver, has shown benefits for dyslipidemia and reductions in weight, fasting insulin, and alcohol use [28, 32, 48]. Early data on FGF-21 analogs, such as pegozafermin and efruxifermin, have demonstrated efficacy in reducing hepatic fat in MASH, as well as improving both steatohepatitis and fibrosis in MASH cirrhosis [28, 32, 48].

### Medical management of metabolic risk factors

Optimal management of CMRFs is of utmost importance in order to reduce cardiovascular risk and all-cause mortality in patients with MASLD and MetALD [32, 48]. In addition to standard management of dyslipidemia, hypertension, diabetes, etc., a number of the medications used to treat CMRFs have been linked to additional liver-specific benefits in MASLD patients [48]. Regarding diabetes management, in addition to the GLP-1 analogs discussed above, sodium glucose co-transporter 2-inhibitors (SGLT2i) have been found to improve hepatic fibrosis and promote weight loss in trials [48, 75]. In patients across the spectrum of CLD, statins may slow progression of hepatic fibrosis to cirrhosis and reduce the risk of key liver-related outcomes including decompensation, HCC, and acute-on-chronic liver failure despite no direct evidence of liver histologic improvement in studies to date [76–78]. Aspirin and other antiplatelet agents may exert similar benefits, possibly through vascular effects [48]. Finally, some studies have linked angiotensin-converting enzyme inhibitors (ACE-I) to a lower risk of cirrhosis-related complications and HCC in MASLD patients, especially in those with chronic kidney disease [75, 79]. Other data suggest that ultimate reduction in cirrhosis-related complications with ACE-Is and angiotensin receptor blockers (ARBs) may occur primarily in the pre-cirrhotic stage through attenuation of hepatic fibrosis; however, even in compensated cirrhosis, these agents have been associated with lower cardiovascular-related mortality [80].

### Approaches to alcohol cessation in MetALD

Another main pillar of MetALD management is alcohol cessation [28]. Not only does alcohol have a direct causal relationship with MetALD, but also, it is known to exacerbate CMRFs, such as hypertension, insulin resistance, obesity, and dyslipidemia [28]. While not all MetALD patients meet the criteria for AUD, alcohol cessation is advised as any reduction in alcohol intake is associated with improved outcomes [48]. The WHO AUDIT-C [81], is the recommended tool for assessing alcohol use [48].

The diagnosis of AUD is complicated by reliance on self-reported alcohol use, and its management is equally challenging, with up to 70% of patients relapsing at some point [28]. Best practices include routine alcohol use screening, patient education, brief motivational interventions, and referral to addiction services when appropriate [48]. Because effective cessation typically requires both psychological and medical support [32], patients should be offered pharmacotherapy in addition to cognitive behavioral therapy [48, 82].

FDA-approved medications for AUD include acamprosate, naltrexone, and disulfiram. Medications for AUD therapy are not only safe and effective but also have been shown to decrease liver decompensation and mortality [82, 83]. Both acamprosate and naltrexone can be used to treat AUD in patients with liver disease characterized as Child-Pugh A or B cirrhosis, although safety data in liver disease are limited [82]. Naltrexone is available as a daily tablet or monthly injection while acamprosate is dosed three times daily [82]. Acamprosate should be dose reduced in those with renal impairment and avoided if creatinine clearance (CrCl) is less than 30 mL/min while naltrexone should be avoided in patients who have recent changes in liver function and in patients taking opioids [82]. Studies have shown that naltrexone and acamprosate improve alcohol-associated outcomes, such as time to relapse and number of drinks per day [83]. In a study of AUD pharmacotherapy in patients with liver disease and AUD, naltrexone and acamprosate had similar survival outcomes; however, similar to prior studies, acamprosate had a slightly lower compliance rate due to its higher daily dose frequency [83]. Disulfiram is not recommended in patients with liver disease given hepatic clearance and risk of hepatic toxicity [48, 82].

Off-label pharmacotherapy options for AUD include baclofen, gabapentin, and topiramate [82]. Baclofen is the only medication studied in patients with cirrhosis and is recommended for the treatment of AUD in ALD; however, it is not recommended for patients with hepatic encephalopathy (HE) and needs to be dose reduced in those with renal disease [82]. Topiramate should also be used cautiously in those with HE given its cognitive side effects and a dose reduction is recommended in those with severe hepatic impairment and CrCl < 70 mL/min [82]. Similarly, gabapentin should also be used with caution in those with cirrhosis and lower initial doses should be considered as it can precipitate HE, and must also be dose reduced in those with kidney disease [82].

### Liver transplant considerations

Indications for liver transplantation in MetALD are similar to other common etiologies of liver disease, and generally include cirrhosis with decompensation, model for end-stage liver disease (MELD) score ≥ 15, and/or HCC within transplant criteria, among others. At present, data addressing practices and outcomes regarding waitlisting and transplant for the MetALD population are limited. Using the UNOS registry, complemented with data from a single center, a recent retrospective cohort study assessed trends in waitlisting and liver transplantation outcomes for this population [39]. For individuals awaiting liver transplantation, those with MetALD experienced a higher rate of waitlist removals, stemming from death or clinical deterioration, and an increase in all-cause mortality and graft failure post-transplant compared to patients with ALD [39]. These observations in the transplant population differ from trends in the non-transplant population where ALD has been associated with higher liver-related morbidity and mortality [24]. It is plausible that MetALD patients who require liver transplant are in a poorer state of health related to the combination of alcohol-associated and CMRFs, in comparison to ALD patients for whom alcohol use alone is the primary driver of disease. Indeed, previous studies in the ALD population have also demonstrated that the presence of diabetes or obesity increases the likelihood of waitlist removal due to death or being considered “too sick” [84]. These findings reinforce the significance of diagnostic accuracy given that MetALD confers worse clinical outcomes in both pre- and post-transplantation settings [39].

Because alcohol-associated and CMRFs act synergistically, the management of MetALD before and after liver transplantation presents unique challenges [85]. Obesity, diabetes, and the potential for alcohol relapse have each been shown to negatively influence long-term survival among transplant recipients [39, 84, 85]. As such, comprehensive management strategies that address both metabolic comorbidities and alcohol use will be essential to improving outcomes in this high-risk population.

**Figure 2:**
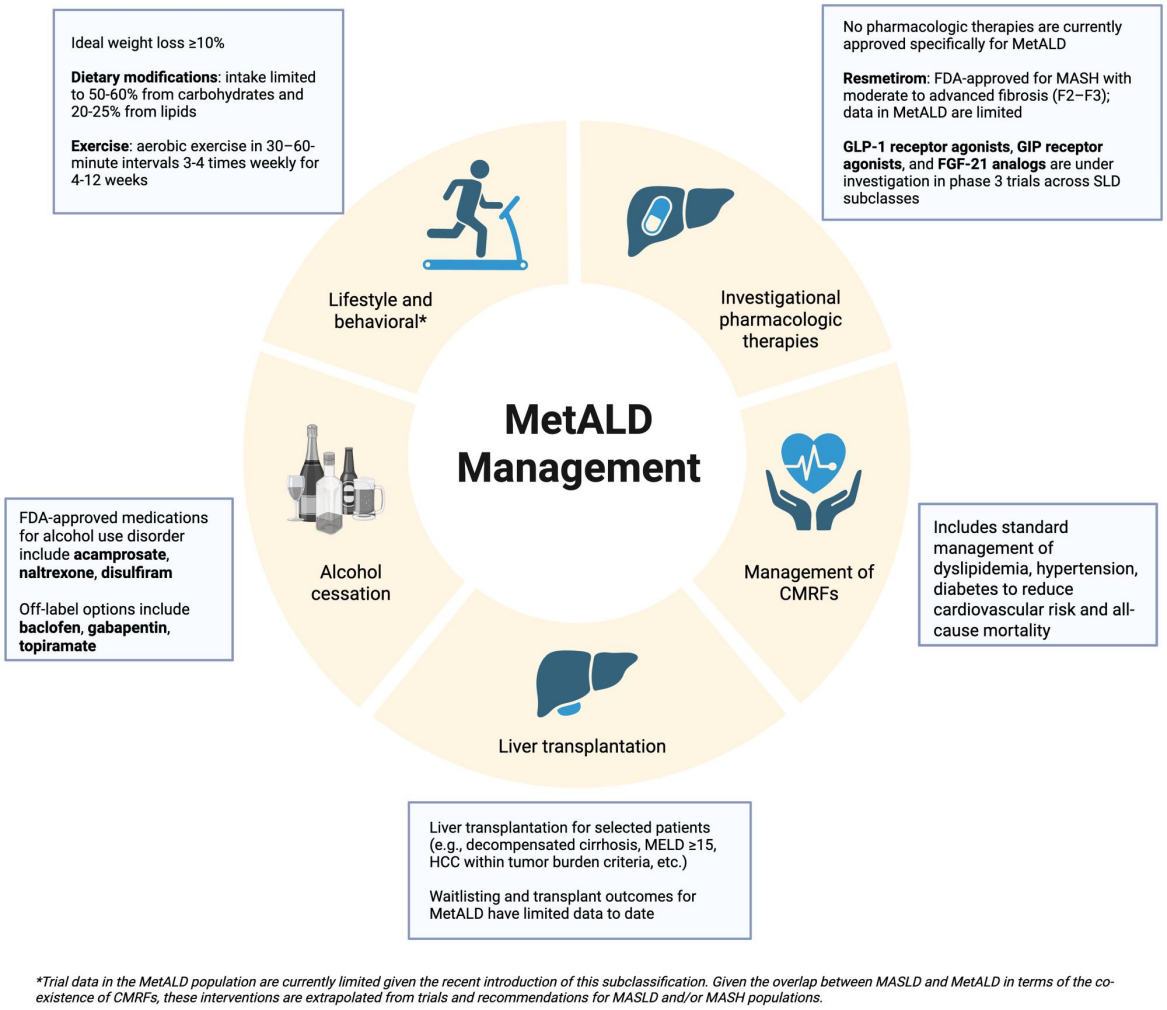
Overview of the management of MetALD. Interventions are aimed at lifestyle and behavioral modifications, investigational pharmacologic therapies, management of CMRFs, alcohol cessation, and the potential role of liver transplantation in selected patients. To date, trial data in the MetALD population is limited. As a result, management options have been extrapolated from trials and recommendations in the MASLD and/or MASH populations, in addition to standard management for alcohol cessation and cardiometabolic risk factors. ALD, alcohol-associated liver disease; CMRFs, cardiometabolic risk factors; FDA, Federal Drug Administration; FGF-21, fibroblast growth factor-21; GIP, gastric inhibitory polypeptide; GLP-1, glucagon-like peptide-1; MASH, metabolic dysfunction-associated steatohepatitis; MASLD, metabolic dysfunction-associated steatotic liver disease; MetALD, metabolic and alcohol-associated liver disease.

An overview of the management options discussed for MetALD, including management of CMRFs, alcohol use, additional lifestyle behaviors, potential pharmacotherapies, and liver transplantation in selected patients, is depicted in Figure 2.

## Implications for research and policy

### Impact of nomenclature changes on research

A central priority during the Delphi process was to preserve the value of existing research on biomarkers and clinical trials, ensuring that the studied populations would not shift substantially with the transition in nomenclature [6, 86]. To date, the transition from NAFLD to MASLD has not produced major disruptions in this regard, with multiple studies supporting the strong diagnostic concordance between the two entities [6, 13].

Although NAFLD and MASLD have distinct definitions, the shared feature of steatohepatitis has enabled clinical trial data from NASH populations to remain applicable to patients now classified under MASLD/MASH [6, 13]. The same holds true for data from biomarker discovery research [6, 13]. Importantly, this continuity applies across diverse patient populations. Data from European, Asian, and Swedish cohorts originally defined as NAFLD continue to be relevant when applying MASLD diagnostic criteria [13, 18, 19, 21]. Most recently, Younossi *et al.* analyzed three decades of tertiary care and national database data, comparing MASLD and NAFLD using non-invasive tests and mortality outcomes [22]. The authors found no significant differences in these metrics, reinforcing that research conducted in the NAFLD populations remains generalizable under the new MASLD designation [22].

### Implications for disease awareness

As part of the nomenclature change process, participants in the Delphi panel were surveyed on the anticipated impact of these changes, with 56% indicating a positive influence on disease awareness [6]. The introduction of the MetALD subclassification is particularly significant, as it formally recognizes a cohort that had not been clearly defined or acknowledged previously. For patients, framing liver disease in terms of both alcohol-related factors and CMRFs facilitated earlier detection and awareness. This framing supports a multi-pronged approach to patient education and care, incorporating both lifestyle interventions and medical management [6, 86].

### Implications for health policy and patient care

As emphasized earlier, there are currently no ICD-10 codes for MetALD, nor are there standardized procedures to reliably assess and track alcohol use within most electronic health record systems (again, with the exception of annual AUDIT-C in the VHA [27, 87]). The absence of standardized coding and measurement poses significant challenges for observational research [32]. Establishing administrative codes for the spectrum of SLD phenotypes is critical for both patient care and research advancement. From a health policy perspective, the lack of standardized coding also has downstream implications for reimbursement, quality measurement, population health surveillance, and equitable access to emerging therapies and clinical trials. Equally important is the development and validation of reliable tools to classify alcohol consumption. For real-world practice, this includes standardized, structured screening instruments integrated into routine care, along with longitudinal documentation of alcohol use patterns over time. In parallel, emerging machine learning and natural language processing approaches may help improve retrospective classification and population-level surveillance, building on applications already demonstrated in areas such as opioid use disorder, traumatic brain injury, and seizure monitoring [88].

A European study identified poor familiarity with MASLD/MASH/MetALD guidelines as a barrier to effective patient care [89]. Similarly, the EASL–Lancet Liver Commission recently underscored the importance of early diagnosis and adherence to guidelines for preventing liver disease [90]. Addressing these gaps requires integrated care models that actively engage primary care physicians in early detection, prevention, and longitudinal management of patients with, or at risk for, MetALD and other SLD phenotypes, supported by policy-level incentives that promote guideline adherence, standardized alcohol screening, and coordinated referral pathways [89]. Public health initiatives should also emphasize campaigns and resources that highlight the associations between alcohol, obesity, and MetALD. Together, these considerations underscore the need for coordinated policy efforts spanning diagnostic coding, alcohol use assessment, guideline dissemination, and public health messaging to ensure accurate identification and management of MetALD across healthcare systems.

### The need for integrated care models

Importantly, the new nomenclature brings appropriate attention to the CMRFs that contribute to MetALD, such as insulin resistance, T2DM, and dyslipidemia [6]. By explicitly acknowledging the synergistic interplay between metabolic and alcohol-associated factors, the updated nomenclature sets the stage for the development of multidisciplinary clinics within both community and hospital settings. In an integrated model, primary care, public health, and specialists from the fields of hepatology, endocrinology, cardiology, and addiction medicine can collaborate to implement interventions that improve health and patient outcomes, both at the individual and population levels [71, 89].

Key Points

•  Prior research conducted in NAFLD remains generally applicable to MASLD populations.
•  The updated SLD nomenclature aims to reduce patient stigma that was associated with former terminology and emphasizes the role of alcohol and CMRFs.
•  There is a need for dedicated administrative diagnostic codes for MetALD.
•  Integrated care models that address the multifactorial nature of SLD will likely improve health outcomes through mitigation of alcohol use and control of CMRFs.

## Future directions

### Variability in alcohol consumption

Although the consensus established quantitative thresholds for daily/weekly alcohol use as part of the triad of diagnostic criteria for MetALD, it does not address the specific duration of alcohol use, or the variability in patterns of alcohol use amongst individuals. As noted previously, alcohol intake often changes substantially over the course of disease. This variability also complicates clinical research, particularly trial design, where post-diagnosis alcohol consumption is a key determinant of prognosis [24]. Incorporating time-varying measures of alcohol exposure and cardiometabolic risk into study design and analysis will be essential to avoid misclassification and to better align trial endpoints with real-world disease trajectories. Future preclinical and clinical trials will also need to account for the potential impact of alcohol on the efficacy of investigational therapies [71]. Future work to establish an effective approach to accurately assess alcohol use in the classification of MetALD will be critical.

### Biomarker discovery

With the continued rise in relevant risk factors, biomarker discovery efforts should prioritize two domains: metabolic status and alcohol use [71]. Numerous validated biomarkers already exist for metabolic dysfunction, including glycosylated hemoglobin, measures of insulin resistance for diabetes, and triglycerides for dyslipidemia. In contrast, accurate assessment of alcohol use remains a major challenge. Self-report questionnaires are subject to bias and may fail to reliably identify individuals with sustained moderate alcohol intake [91]. Misclassification is not uncommon, as several studies have shown patients labeled with MASLD in fact met criteria for MetALD, with implications for both treatment and prognosis [40, 41, 71]. While biomarkers, such as urinary PEth and EtG, can detect alcohol use, there is a pressing need for novel, cost-effective, sensitive, and specific biomarkers capable of quantifying both the level and pattern of alcohol consumption, representing an area with considerable promise for future research [71, 91].

### Longitudinal phenotyping and disease trajectories

Given the dynamic nature of alcohol consumption and cardiometabolic risk, future studies should prioritize longitudinal phenotyping approaches that explicitly model transitions between MASLD, MetALD, and ALD over time. Rather than treating SLD subclasses as static diagnoses, trajectory-based frameworks may better capture real-world disease evolution and allow more accurate prediction of clinically meaningful outcomes, including hepatic decompensation, cardiovascular events, and mortality. Such approaches will also be essential for evaluating the impact of sustained alcohol reduction or metabolic improvement on disease progression and reversibility.

Together, these methodological and conceptual advances provide a framework for refining both research and clinical care in MetALD. Looking ahead, future work should aim to bridge the gap between self-reported alcohol consumption and objective measures to more accurately diagnose and manage the spectrum of SLD. Randomized clinical trials specifically focused on the MetALD population are needed to clarify the applicability of existing MASLD and ALD guidelines, while also driving investigation into novel pharmacologic therapeutics tailored to this group. In parallel, implementation-focused research will be needed to determine how best to operationalize MetALD classification, alcohol assessment, and integrated care models across diverse healthcare systems.

## Conclusion

The updated nomenclature and the conceptualization of MetALD as a class under the umbrella of SLD acknowledges the synergistic relationship between alcohol and CMRFs as key drivers of liver disease. The adoption of this nomenclature also underscores the multifaceted and dynamic nature of SLD classes, reduces the stigma associated with the former terminology, and offers new avenues of investigation. Clinicians should aim to provide multidisciplinary care with a focus on alcohol cessation and management of metabolic comorbidities using a combined approach of lifestyle changes, psychological resources, and medication therapy. The implications and challenges that stem from the novel nomenclature warrant further research into the accurate assessment, therapeutic interventions, and policies surrounding MetALD and liver disease.
